# Factors Affecting Mass Transport Properties of Poly(ε-caprolactone) Membranes for Tissue Engineering Bioreactors

**DOI:** 10.3390/membranes8030051

**Published:** 2018-08-01

**Authors:** Nazely Diban, Beatriz Gómez-Ruiz, María Lázaro-Díez, Jose Ramos-Vivas, Inmaculada Ortiz, Ane Urtiaga

**Affiliations:** 1 Department of Chemical and Biomolecular Engineering, University of Cantabria, Avda. Los Castros s/n, 39005 Santander, Spain; beatriz.gomezruiz@unican.es (B.G.-R.); ortizi@unican.es (I.O.); urtiaga@unican.es (A.U.); 2 Servicio de Microbiología, Hospital Universitario Marqués de Valdecilla e Instituto IDIVAL, 39005 Santander, Spain; marialazarodiez@gmail.com (M.L.-D.); jvivas@idival.org (J.R.-V.); 3 Red Española de Investigación en Patología Infecciosa (REIPI), Instituto de Salud Carlos III, 28029 Madrid, Spain

**Keywords:** membrane fouling, membrane plasticization, nutrients transport properties, perfusion bioreactors, tissue engineering

## Abstract

High porosity and mass transport properties of microfiltration polymeric membranes benefit nutrients supply to cells when used as scaffolds in interstitial perfusion bioreactors for tissue engineering. High nutrients transport is assumed when pore size and porosity of the membrane are in the micrometric range. The present work demonstrates that the study of membrane fouling by proteins present in the culture medium, though not done usually, should be included in the routine testing of new polymer membranes for this intended application. Two poly(ε-caprolactone) microfiltration membranes presenting similar average pore size (approximately 0.7 µm) and porosity (>80%) but different external surface porosity and pore size have been selected as case studies. The present work demonstrates that a membrane with lower surface pore abundance and smaller external pore size (approximately 0.67 µm), combined with adequate hydrodynamics and tangential flow filtration mode is usually more convenient to guarantee high flux of nutrients. On the contrary, having large external pore size (approximately 1.70 µm) and surface porosity would incur important internal protein fouling that could not be prevented with the operation mode and hydrodynamics of the perfusion system. Additionally, the use of glycerol in the drying protocols of the membranes might cause plasticization and a consequent reduction of mass transport properties due to membrane compaction by the pressure exerted to force perfusion. Therefore, preferentially, drying protocols that omit the use of plasticizing agents are recommended.

## 1. Introduction

The production of engineered artificial 3D tissues and bioartificial organs is the utmost challenging aim of tissue engineering. Important applications of functional bioartificial 3D tissues are therapeutics as, for example, bioartificial organs for transplantation, or for ex vivo treatment and in vitro models for research on diagnosis tools and/or pharmaceuticals. Bioreactors aid the in vitro generation of 3D artificial tissues, although maintaining their required structural integrity and functionality is difficult [1]. Furthermore, the transport of oxygen and nutrients to artificial tissues and neovascularization of the living tissue are still important limitations [2,3]. 

Membrane perfusion bioreactors providing interstitial flow of the culture media might help overcome these limitations [3,4]. The importance of hydrodynamics on the performance of nutrients supply in bioreactors has been largely proven and many research studies aim at understanding and improving the fluid dynamics of different bioreactor designs [5,6]. Furthermore, the membrane used as scaffold support for cells’ proliferation and differentiation represents an important barrier for the successful access of nutrients to the cells. Literature usually focuses on theoretical analysis to model the effect of flow dynamics within 3D scaffolds in bioreactors [7,8]. Most of these studies analyze the effect of flow conditions on flow patterns and shear stresses to the cells without taking into consideration the porosity or transport through the scaffolds [8,9]. Simulations of interstitial perfusion bioreactors for cartilage regeneration have evidenced the outstanding benefits that provide the conjunction of the improved nutrients accessibility and catabolites removal from the cell compartment, and the mechanic-biological stimuli produced by the shear stress of the flow on the cell membrane [10].

A wide range of literature can be found on the fabrication of novel and enhanced artificial membranes for cell proliferation and differentiation [4,11]. The characterization of properties of these novel materials is usually focused on the morphological aspects and membrane-cell interactions, but little effort has been paid to the experimental assessment of the nutrients transport performance under dynamic fluid conditions of the new materials and membranes aimed at tissue engineering scaffoldings. Transport properties of membrane scaffolds under steady-state conditions have been scarcely studied [12,13,14,15,16]. Alternatively, the accessibility of the nutrients to the cells could be extrapolated by the porosity of the membrane, assuming that high porosity involves high nutrients permeability. However, Wang et al. [17] realized that in other works this assumption was not always supported by experimental evidence. They pointed out the interest in experimentally quantifying permeabilities of the scaffolds as a performance parameter of the nutrients and metabolites transport properties. The study of the transient flux properties (compaction and membrane fouling) of micro- and ultrafiltration membranes that is well-known and widely reported in the literature related to biotechnology, food, beverages, etc. [18], has been omitted for perfusion membrane bioreactors. Membrane compaction is usually caused by a mechanical deformation of the membrane under pressure that would change the porous structure [19] and consequently the actual cell-substrate interactions. The change in the porous structure would also affect the effective nutrients transport properties as well as the fouling mechanisms occurring on the membrane. Prior to investing efforts in evaluating morphological and cell-substrate interactions, it seems reasonable to preliminarily evaluate transient phenomena on newly fabricated membrane scaffolds to establish a more realistic idea about their viability in bioreactors for tissue engineering. Furthermore, this preliminary analysis can be used to further design and optimize the hydrodynamic configuration of perfusion bioreactors that incorporate such membrane scaffolds.

Two different poly(ε-caprolactone) (PCL) microporous membranes have been previously fabricated [20], reporting good morphology and promising cell adhesion and proliferation properties, to be used as scaffolds for tissue engineering. Aside from subtle morphological differences, no conclusive evidence was found to discriminate one membrane type from another. The present work aimed at evaluating the functional nutrients transport performance of these PCL membranes as a scaffold within an interstitial perfusion membrane bioreactor. The transient mass transport properties of the membranes under dynamic flow rate conditions were assessed by measuring the behavior of the flux of water and aqueous solutions of the model protein bovine serum albumin (BSA) through the above-mentioned PCL membranes. The effect of flow configuration, temperature and pH of the feed stream on the fouling and steady-state transport properties of the membranes was addressed. Additionally, the influence of the post-treatment protocols followed during the fabrication process for the storage of dry membranes on their transport properties was explored.

## 2. Materials and Methods 

### 2.1. Membrane Preparation

Flat PCL membranes were prepared by phase inversion reproducing the procedure described elsewhere [20,21]. Briefly, PCL (MW 80 kDa, Sigma Aldrich, Madrid, Spain) polymer solution of 15% *w*/*w* was prepared by dissolving PCL under stirring in *N*-methylpyrrolidone (NMP, 99% purity, Acros Organics, Madrid, Spain) at 35 °C for 24 h, then filtered through a 25-µm metallic mesh filter under pressure and left to degas overnight. The polymer solution was cast at room temperature on a glass plate using a 0.2 mm-thickness casting knife and immediately submerged into the coagulation bath composed of either ethanol (EtOH, technical grade, Acros Organics) or 2-propanol (IPA, 99% purity, Oppac, Navarra, Spain). After complete polymer coagulation and solvent traces removal by a washing procedure [20], different post-treatment protocols were employed: (i) The membranes were immersed in a solution of 25% *v*/*v* glycerol/water and further air dried and stored, and (ii) the membranes were maintained wet submerged in water until characterization. Membranes stored dry were submerged in ultrapure water for 24 h before filtration tests.

Several repetitions of the casting/coagulation procedure were undertaken and representative membrane samples were tested. Depending on the coagulation bath (EtOH and IPA) and the post-treatment protocol (glycerol (Gly) and wet) the membranes were designated with membrane code M-EtOH/Gly, M-EtOH/wet, M-IPA/Gly and M-IPA/wet, respectively. 

### 2.2. Water and Protein Flux

Clean water flux (CWF) and volumetric flux of BSA solutions were evaluated using two filtration configurations: (i) Dead-end or normal flow filtration (NFF), and (ii) tangential flow filtration (TFF). The NFF experiments were undertaken in a commercial Amicon stirred cell (Millipore, Madrid, Spain) with 13.4 cm^2^ effective filtration area, *A_e_*, and were described in detail elsewhere [20]. The TFF system consisted of a 500 mL feed tank connected to a peristaltic pump (Minipuls 3, Gilson, Madrid, Spain) that fed the fluid tangentially to a membrane rectangular cell of *A_e_* = 10 cm^2^. The retentate was recirculated to the feed tank. In both systems, the feed was formed either by ultrapure water for CWF experiments, or by a model protein solution, BSA (lyophilized powder, ≥96% (agarose gel electrophoresis), Sigma Aldrich) at a concentration of approximately 0.4 g/L, either in ultrapure water or in a phosphate buffer solution (PBS) at pH 7.2. Although the pH of the BSA solution in water was not controlled, it was measured during the experiments, observing a value of approximately 5. Before filtration, membranes were pre-soaked in water for 24 h. The experiments were done either at room temperature or at 37 °C.

The fluid permeated through the membrane was collected at a constant transmembrane pressure, Δ*P*, in the range 0.1–0.4 bar. The volumetric fluid flux (*Jv* [L m^−2^ h^−1^]) was calculated at each transmembrane pressure considering the effective filtration area for each filtration system. The overall permeance of the scaffold, *K_T_*, was determined as follows:

*K_T_* [L m^−2^ h^−1^ bar^−1^] = *Jv*/Δ*P*, (1)

The BSA concentration in the permeate (*C_BSA,p_*) and in the feed tank (*C_BSA,F_*) was measured by UV-vis spectroscopy (UV-1800, Shimadzu, Duisburg, Germany) at 280 nm. The percentage of BSA permeated through the membrane or BSA transmission (*T_BSA_*) was calculated as detailed in equation 2:

*T_BSA_ [%]* = *C_BSA,p_*/*C_BSA,F_* × 100, (2)

At least three replicates of the flux experiments were undertaken. The values were expressed as the average ± standard deviation.

Confocal Laser Scanning Microscopy (CLSM) technique was used to take images of the surface and cross section of the membranes after BSA filtration experiments. The membranes were stained with 200 µL of FilmTracer™ SYPRO^®^ Ruby Biofilm Matrix (Invitrogen, Carlsbad, CA, USA), incubated in the dark for 30 min at room temperature, and rinsed with distilled water. As control samples, membranes after water filtration were used. Microphotographs were obtained with a Nikon A1R confocal scanning laser microscope (Barcelona, Spain). A 405 nm-excitation, 662–737 nm-emission filter was used. Images were captured at random with a ×20 Plan Apo 0.75 NA objective.

### 2.3. Membrane Morphology (Physical Characterization)

Surface morphology of the membranes was evaluated with scanning electron microscopy (SEM, EVO MA 15, Carl Zeiss, Weimar, Germany) at a voltage of 12.6 kV. Samples were gold sputtered before SEM analysis.

Pore size and pore size distribution of the membranes were measured by liquid extrusion porometry technique using capillary flow porometer (Porolux 1000, IB-FT GmbH, Berlin, Germany) and Coulter Porometer II (Coulter Electronics Limited, Luton, UK). 

### 2.4. Statistical Data Treatment

The statistical data analysis of significance of the differences in the properties between the two experimental populations (e.g., two membrane types M-EtOH and M-IPA or between two types of filtration configurations) (*n* ≥ 3), were evaluated using the *t*-test for unequal variances (*p* < 0.05). The significance of the properties’ differences among more than two populations (e.g., M-EtOH (NFF), M-EtOH (TFF), M-IPA (NFF) and M-IPA (TFF)) was determined using one-way ANOVA with Bonferroni post hoc analysis (*p* < 0.05).

## 3. Results

### 3.1. Protein Fouling

**Figure 1:**
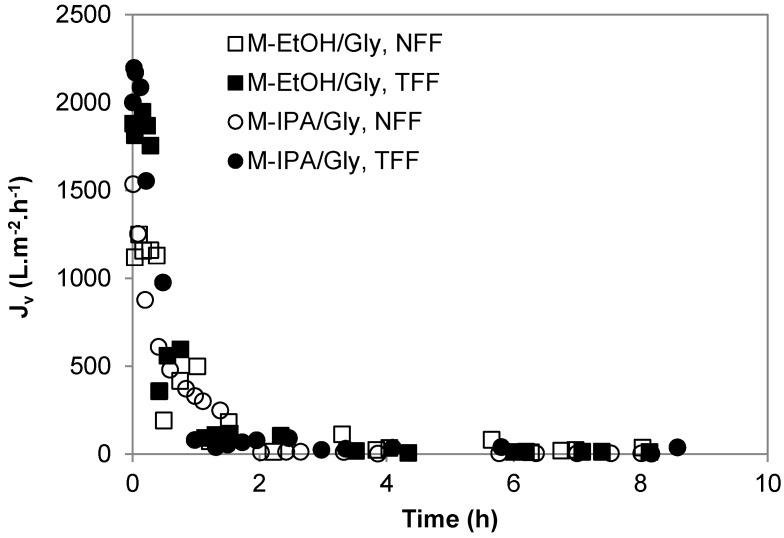
Representative curves of the change with time of the overall flux of M-EtOH/Gly and M-IPA/Gly membranes during BSA filtration experiments under NFF and TFF configurations at a constant working pressure of 0.2 bar, at room temperature and pH ~ 5.

Figure 1 depicts representative curves of the change with operation time of the overall volumetric flux permeated through the M-EtOH/Gly and M-IPA/Gly membranes in NFF and TFF configurations at room temperature and pH 5. Continuous flux decay can be observed during BSA filtration. The overall flux (Figure 1) and BSA permeances at steady-state (Table 1) of M-EtOH/Gly and M-IPA/Gly membranes presented not significantly different values independently of the filtration mode used. The steady-state water permeances of M-EtOH/Gly and M-IPA/Gly membranes (Table 1) fall within the range of the values reported previously for polymeric membranes for tissue engineering (from 20 L m^−2^ h^−1^ bar^−1^ [22] to 2100 L m^−2^ h^−1^ bar^−1^ [23]). However, the comparison of the overall average CWF and BSA solution membrane permeances (*K_T_*) in Table 1 demonstrated that they were significantly lower for BSA filtration than for clean water filtration, with the exception of the M-EtOH/Gly membrane working under TFF mode that had comparable permeances for clean water and BSA solutions filtration. Wang et al. [17] also observed that protein solution permeability was three times lower than that of water through microporous PCL tubular scaffolds. In general, the permeance decline of BSA solutions through M-IPA/Gly membranes in comparison with water permeance was higher in NFF operation mode than in TFF mode (Table 1). This might be attributed to the favorable hydrodynamics in TFF mode to avoid concentration polarization effects and cake formation on the surface of microfiltration membranes by biomolecules (e.g., proteins, polysaccharides, microorganisms, etc.) [24,25,26]. 

The average results of BSA transmission presented in Table 1 show that during the operation time the values were always above 90%. The difference of *T_BSA_* values reported in Table 1 was not significant. The BSA molecular size (67 kDa, ~10 nm) is far smaller than the membrane pore size (Table 1), belonging to the microfiltration range, and 100% BSA transmission should be expected. However, Persson et al. [26] also reported similar BSA transmission values through polyether sulphone and nylon membranes during cross-flow microfiltration (with the same BSA concentration and pH (0.4 g/L and pH 5) and similar transmembrane pressures to those used in the present work (0.05 and 0.12 bar)). This behavior was attributed to fouling caused by BSA aggregates easily formed at the isoelectric point of the protein (4.7–4.9 for BSA). 

**Figure 2:**
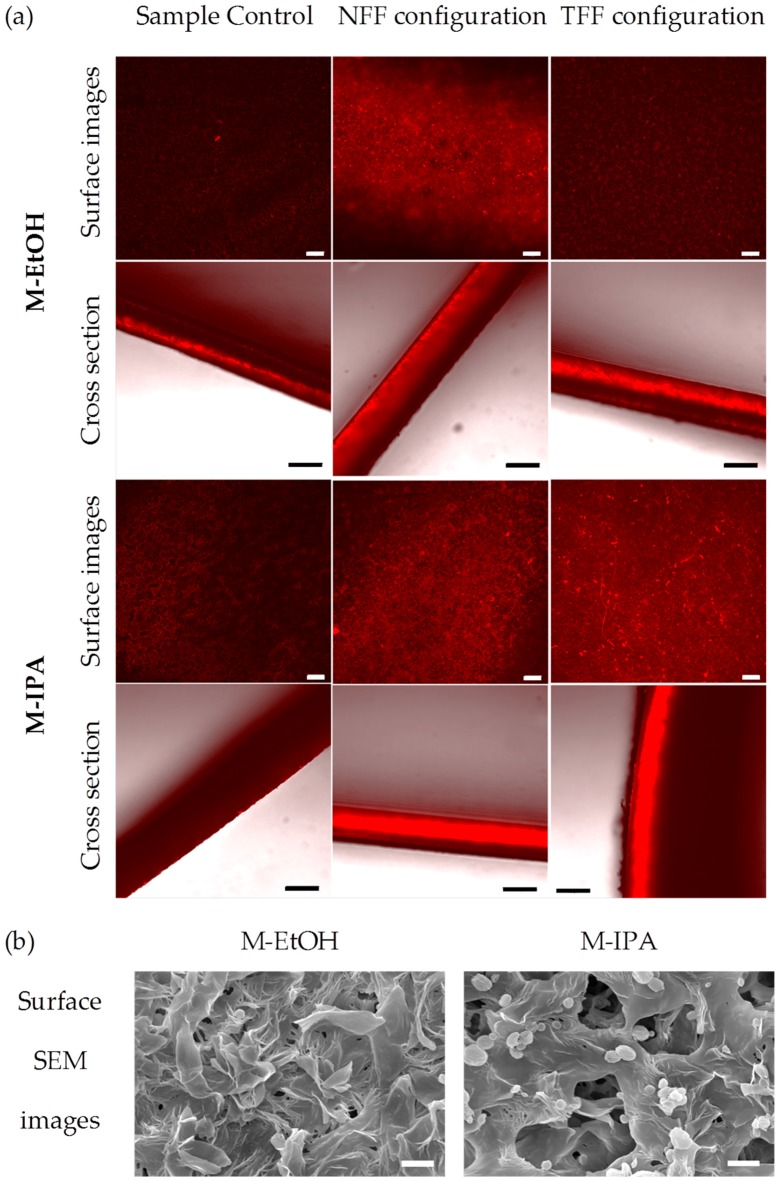
(**a**) Confocal of the surface (scale bar 50 µm) and cross section (scale bar 100 µm) and (**b**) surface SEM images (scale bar 3 µm) of the M-EtOH and M-IPA membranes. Red fluorescence indicates protein presence in confocal images. Confocal control images are used to evaluate the efficacy during the dye washing and any possible membrane auto fluorescence.

Figure 2a shows CLSM images for the surface and cross section of the membranes. Red fluorescence indicates protein presence. Control images are presented for comparison. The images show a strong influence of the filtration configuration and the membrane type on protein presence and fouling mechanisms. M-EtOH presents mainly external fouling (in the membrane surface) when NFF is used, while for TFF the surface of the membrane presented less fluorescence intensity. In the case of M-IPA, both surface and cross section showed important protein presence, indicating that both internal and external fouling mechanisms were present independently of the filtration configuration. These results are in agreement with the surface pore morphology shown in Figure 2b and with the experimental results of overall permeance discussed above. The decline of the overall permeance of the BSA solution in TFF configuration was more important for M-IPA than for M-EtOH, which remained similar to the overall permeance of clean water solutions. The surface roughness and porosity has been proven an important parameter to explain the mechanisms of protein fouling in microfiltration membranes [26,27,28]. It has been found that membranes with high surface roughness might suffer faster fouling [26]. In previous works [20], the average pore size of the surface and the average surface roughness, *Ra*, were found to be 0.67 ± 0.48 µm and 617 ± 19 nm for M-EtOH, and 1.70 ± 0.62 µm and 678 ± 8 nm for M-IPA. Also, although the overall porosity was similar for both membranes (about 85%), the presence of pores on the membrane surface was much more abundant for M-IPA membranes than for M-EtOH, as can be seen in Figure 2b. Therefore, M-EtOH has a smooth surface roughness with scarce pores of small size that prevented BSA infiltration in the membrane structure and facilitated the surface cleaning by the shear flow rate in TFF. The more probable mechanism of fouling for M-EtOH was, therefore, a cake formation of BSA that could be easily swept under TFF mode. On the other hand, M-IPA surface morphology and pore size allowed the internal fouling by the BSA infiltration into the membrane structure that TFF configuration could not avoid.

In order to simulate the temperature and pH conditions to be used during dynamic culture in bioreactors, experiments of CWF and BSA model solution using PBS at pH 7.2 and at 37 °C were done using M-IPA/Gly membranes under TFF mode. Overall permeance values at steady states of 244 ± 50 L m^−2^ h^−1^ bar^−1^ and 110 ± 30 L m^−2^ h^−1^ bar^−1^ for CWF and BSA solution filtration tests, respectively, were achieved (Table 1). From these results we can conclude that temperature had a slight enhancing effect on CWF permeance from 218 to 244 L m^−2^ h^−1^ bar^−1^ although was not statistically significant. On the other hand, at 37 °C and a pH of 7.2, the permeance of BSA solution through M-IPA/Gly membranes under TFF mode was not altered. Therefore, the expected improvement of temperature on flux values was somehow limited by the pH effect. Moreover, *T_BSA_* was reduced to 76 ± 3%. Pierson et al. [26] reported a reduction in the total flux and BSA transmission of polyethersulphone microfiltration membranes at pH 7 in contrast to pH 5. At pH 5, BSA is in the proximity of the isoelectric point, and therefore neutral charge and low repulsion between BSA molecules would facilitate the formation of large aggregates of BSA molecules in the internal porosity of M-IPA membranes. These large aggregates would produce large pores in the fouling cake facilitating BSA transmission. At pH 7, both PCL and BSA present negative charges and therefore BSA molecules are less prone to aggregate and would cause a reduction of the effective pore size of the internal fouling cake. This is in agreement with the Atomic Force Microscopy (AFM) images after filtration of BSA solutions reported by Huisman et al. [29] for polysulphone ultrafiltration membranes with nominal cut-off of 300 kDa. In these low-retentive ultrafiltration membranes, the flux properties were mainly governed by the fouling layer structure that, both at pH 5 and 7, importantly reduced the effective porosity of the membrane after BSA filtration; however, the effective pore size of fouled membranes at pH 7 was much smaller than at pH 5. Additionally, at pH 7, both PCL membrane and BSA surfaces are negatively charged, which may also increment membrane-protein electrostatic repulsion and the consequent transmission decay [26].

Examining these results, the positive effect on flux properties of M-IPA membranes at higher temperature would be compensated by the reduction on flux properties at pH 7 and, therefore, the flux properties for the M-IPA and M-EtOH membranes reported at room temperature and pH 5 could be assumed to be representative of those expected at 37 °C and pH 7, with the exception of protein transmission that is slightly lower, though still sufficient, for cell culture. 

### 3.2. Transient Membrane Compaction

**Figure 3:**
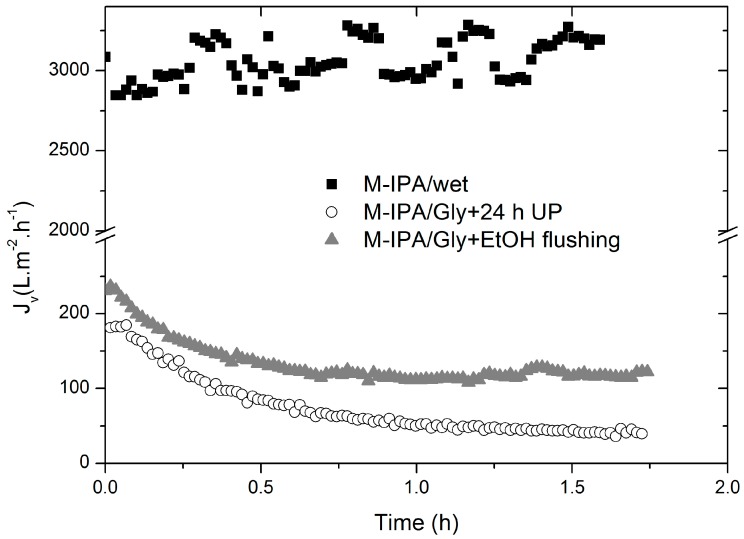
Representative curves of the change with time of the overall water flux of M-IPA/Gly and M-IPA/wet membranes during CWF filtration experiments under TFF mode at a constant working pressure of 0.16 bar and 37 °C.

During the CWF filtration tests of M-EtOH/Gly and M-IPA/Gly membranes, a flux decline was observed, either at room temperature or 37 °C, that could not be attributed to protein membrane fouling. Furthermore, contrary to the CWF decline observed in the present membranes, previous work [21] observed a fast stabilization of water flux for similar flat PCL membranes fabricated by phase inversion technique using EtOH and IPA as coagulants, and much higher water permeances at steady-state were attained (18,428 and 24,772 L m^−2^ h^−1^ bar^−1^, for M-EtOH and M-IPA, respectively). Comparing the experimental procedure of membrane fabrication by Diban and Stamatialis [21] and the present work, the authors did not report any drying protocol or the use of any preserving solution for the membranes [21]. Therefore, it was decided to evaluate CWF transport properties of pristine M-IPA/wet membranes under TFF filtration mode and compare with M-IPA/Gly membranes (Figure 3).

A drastic decline of the initial CWF value was observed from approximately 3000 L m^−2^ h^−1^ for M-IPA/wet to 180 L m^−2^ h^−1^ for M-IPA/Gly membranes. Moreover, M-IPA/wet membranes maintained a constant CWF around 3000 L m^−2^ h^−1^ during more than 1.5 h of operation. These results are in agreement with the values reported by Diban and Stamatialis [21] for M-IPA membranes. In contrast, M-IPA/Gly membranes presented a progressive flux decline similar to what was observed in the previous section. Experiments were reproduced using the same TFF system and following the same protocols for M-IPA/wet membranes and M-IPA/Gly membranes; therefore, the possible fouling coming from Milli-Q impurities could be discarded. As previously indicated, M-IPA/Gly membranes were only submerged 24 h in Milli-Q water in order to remove glycerol from the membrane pores before CWF. To ensure that the flux decline could not be attributed to remaining glycerol in the membrane, a thorough cleaning protocol using 50% *v*/*v* EtOH/Milli-Q water for 24 h followed by a membrane flushing with 50% *v*/*v* EtOH/Milli-Q water at 0.16 bar for 1 h was used. Afterwards, CWF tests were performed (see M-IPA/Gly + EtOH flushing data in Figure 3). The initial water flux observed was not significantly improved with respect to M-IPA/Gly membranes cleaned only with Milli-Q water for 24 h. In addition, a progressive flux decline with time was observed, although a slight improvement of the final flux at steady state was observed from 50 L m^−2^ h^−1^ to 110 L m^−2^ h^−1^ when the cleaning protocol with EtOH was used.

Examining these results, the flux attenuation observed in M-IPA/Gly membranes could be only attributed to a compaction effect or a mechanical deformation of the membrane during the filtration experiments as the membranes are subjected to pressure. Therefore, the membrane pore size would shrink progressively, leading to a consequent flux decline [19,30]. This behavior could be explained by the well-known plasticizing effect that glycerol produces on polymeric membranes [31]. The plasticizing effect of glycerol reduces the intermolecular forces, increasing the mobility of the biopolymer chains, and causes important modification of the mechanical properties of the materials. The presence of glycerol on chitosan films was reported to decrease the tensile strength and enlarge the extension at break in contrast to pristine chitosan films [31,32,33]. From our previous results (Figure 3), the modification of mechanical properties of the M-IPA membranes was permanent even after thorough glycerol washing. Additionally, when the membranes without glycerol were allowed to dry and tested, no flux was detected. This effect could be attributed to the collapse of the porous structure during membrane drying. 

## 4. Discussion

In addition to the adequate morphological and cell-substrate interaction parameters commonly investigated for newly fabricated membranes aimed at being used as scaffolds for tissue engineering, possessing good nutrients transport properties is critical for the success of their applications in membrane interstitial perfusion bioreactors. The behavior of the membranes during the transient stage at the beginning of the operation time in filtration experiments might provide some hints about their suitability for that application. The appearance of compaction and/or fouling phenomena on the membranes under pressure-driven filtration conditions might cause reversible or irreversible subtle changes to the membrane morphology that would alter the effective nutrients transport properties and ultimately affect the cell-membrane interactions. These transient phenomena could be observed under dynamic culture conditions. The present work aims at evaluating the mass transport properties of two PCL flat membranes (M-EtOH and M-IPA) previously characterized [20] in order to establish their adequacy as scaffolds in interstitial perfusion bioreactors for tissue engineering. 

To be successfully used in cell proliferation and differentiation bioreactors, the membranes used as scaffolds must provide sufficient nutrients supply. Nutrients (glucose, proteins, amino acids, vitamins, etc.) are present in the culture medium. Among the different nutrients present in the culture medium for cell sustenance, the most abundant and important for cell metabolism are glucose and BSA protein; in particular, BSA is the largest molecule present in the serum supplemented culture medium. Furthermore, many studies focus on the membrane fouling by BSA as a model protein to study the traditional mechanisms of membrane fouling, even for microfiltration membranes, due to its high molecular size and adsorptive affinity towards polymer materials [26,27,34,35]. Although M-EtOH and M-IPA membranes have a mean pore size in the order of microfiltration membranes (average 0.7 µm), fouling by proteins has been also reported on these types of membranes. Therefore, it is deemed that the major contribution to any fouling mechanism occurring in macroporous membrane scaffolds in contact with culture media could be attributed to BSA deposition. Consequently, BSA filtration experiments were carried out to evaluate the possible BSA deposition on the membranes and/or pore obstruction that could hamper nutrients’ availability to the cells. 

Under steady-state conditions both membranes presented average total permeances of BSA model solutions between 40 and 250 L m^−2^ h^−1^ bar^−1^, considered acceptable for tissue engineering applications. The membranes were tested under two different flow configurations, normal and tangential flow filtration, observing that the flow configuration was important regarding protein fouling effects. TFF mode reduced the fouling effect in comparison to NFF mode, as external protein cake was removed. The comparison between M-EtOH and M-IPA membranes evidenced the important role of surface pore size and porosity of the membranes on the fouling mechanism. Thus, M-EtOH membranes with much smaller surface pore size and porosity could prevent internal protein fouling and thus TFF mode could avoid completely the external fouling effect, obtaining similar flux values as when clean water was filtrated. The porous structure of the membranes depends on the processing variables used during phase inversion. Thus, a thorough study on different combinations of solvents/coagulants for the membrane processing would help the production of optimal membrane porosity to limit protein fouling. The experiments simulating bioreactor temperature and pH conditions presented similar flux values to those at room temperature and pH 5, due to a compensating effect between both variables. Therefore, experiments of M-EtOH and M-IPA membranes at room temperature could be considered representative of the expected behavior of these membranes under bioreactor conditions. The reduction in BSA transmission from 91% at room temperature and pH 5, to 76% at 37 °C and pH 7, was explained by the reduction of effective pore size of the membrane caused during the internal fouling. It must be noted that the high fouling observed on M-EtOH and M-IPA membranes can also be attributed to the high hydrophobic character of PCL that might favor protein adherence [36,37]. The adsorption of BSA and mainly fibrinogen on PCL substrates has shown a favorable effect on the homogenous distribution and attachment of human primary endothelial cells compared to plain PCL surfaces with no proteins adsorbed [36]. Therefore, though the surface modification of PCL membranes to make them more hydrophilic would reduce BSA fouling effect, this could also reduce protein adhesion and hamper cell attachment.

The study of the transient behavior of the membranes indicated that both membranes present a strong compaction effect, with a flux decline above 84% both in normal and tangential flow filtration. Experimental evidence pointed at the use of glycerol solution, used to preserve the porous structure after air drying, to be the cause of this drastic loss of flux membrane performance. Due to the biodegradable characteristics of PCL, storage in water is not recommendable. Therefore, these results demonstrated that an adequate drying protocol should be undertaken, substituting the plasticizing agent (glycerol) for either a solvent-exchange method, to reduce progressively the surface tension of water, or freeze-drying, to preserve the integrity of the porous structure of the PCL membranes after drying while additionally maintaining their intrinsic nutrient transport properties.

## 5. Conclusions

In conclusion, when novel polymer membranes with pores in the rage of microfiltration are prepared to be used as scaffolds in perfusion bioreactors for tissue engineering, in addition to the characterization of morphological and biological cell-membrane interactions, important attention should be paid to the functional transport properties to evaluate their suitability for the intended application. Protein fouling is a phenomenon appearing to be unavoidable in a bioreactor system for cell culture; however, altering the surface membrane pore size and porosity, by means of controlling the membrane fabrication variables and the hydrodynamic perfusion mode of the bioreactor, could prevent or reduce this effect to ensure sufficient nutrients supply to the cells. 

Nevertheless, the results of this work suggest that careful attention should be paid to the preserving protocol used when preparing these membranes. In order to preserve porous microstructure during drying of highly porous membrane scaffolds, the use of plasticizing agents should be avoided, as they may have a counterproductive effect on mechanical properties and a consequent reduction on transport properties when the culture medium is forcedly perfused by pressure in the bioreactor. Instead, other drying procedures should be tested, such as low surface tension solvents exchange or freeze-drying.

## Figures and Tables

**Table 1:** Flux parameters (overall permeance at the steady-state (s.s.) for CWF and BSA filtration, *K_T_*, and BSA transmission (*T_BSA_*)) in dependence the filtration configuration mode (NFF or TFF), temperature and BSA solution pH and membrane pore size (MFP, BPP and SP)) of M-EtOH/Gly and M-IPA/Gly membranes. The statistical differences of the average values of *K_T_* and *T_BSA_* were compared among the groups M-EtOH/Gly, NFF; M-EtOH/Gly, TFF; M-IPA/Gly, NFF and M-IPA/Gly, TFF. MFP, BPP and SP were compared between M-EtOH/Gly and M-IPA/Gly membranes, independently of the filtration configuration. The statistical data analysis showed no significant differences (*p* < 0.05) among the samples. * indicated significant difference of BSA steady-state permeance vs CWF steady-state permeance using the *t*-test for unequal variances (*p* < 0.05).

Parameter	Room T and BSA Solution pH ~ 5				37 °C, BSA Solution pH 7.2
	M-EtOH/Gly, NFF	M-EtOH/Gly, TFF	M-IPA/Gly, NFF	M-IPA/Gly, TFF	M-IPA/Gly, TFF
*K_T_*, s.s. (L m^−2^ h^−1^ bar^−1^) for CWF	209 ± 27	160 ± 77	309 ± 90	218 ± 33	244 ± 50
*K_T_*, s.s. (L m^−2^ h^−1^ bar^−1^) for BSA solution	99 ± 14 *	165 ± 82	46 ± 14 *	116 ± 50 *	110 ± 30 *
*T_BSA_*(%)	98 ± 3	91 ± 11	93 ± 6	91 ± 10	76 ± 3
Mean Flow Pore Size, MFP (µm)	0.72 ± 0.17		0.74 ± 0.17		
Bubble Point Pore Size, BPP (µm)	1.06 ± 0.39		0.97 ± 0.26		
Smallest Pore Size, SP (µm)	0.53 ± 0.20		0.58 ± 0.17		
